# Mini-Mental State Examination and proton spectroscopy of the posterior cingulate in Alzheimer disease

**DOI:** 10.1590/S1980-57642008DN10300005

**Published:** 2007

**Authors:** Hae Won Lee, Paulo Caramelli, Maria Concepcion Garcia Otaduy, Ricardo Nitrini, Claudia da Costa Leite

**Affiliations:** 1 Department of Radiology, University of São Paulo School of Medicine.; 2 Department of Neurology, University of São Paulo School of Medicine and Department of Internal Medicine, University Federal of Minas Gerais, Belo Horizonte.; 3 Department of Neurology, University of São Paulo School of Medicine; Behavioral and Cognitive Neurology Unit, Department of Neurology, and Cognitive Disorders Reference Center (CEREDIC). Hospital das Clínicas of the University of São Paulo School of Medicine.

**Keywords:** proton spectroscopy, posterior cingulate, single voxel, MMSE test, Alzheimer disease

## Abstract

To compare metabolite ratios in the posterior cingulate with the Mini-Mental State Examination (MMSE) test scores in patients with mild or moderate Alzheimer disease and in controls.

**Methods:**

We evaluated 29 patients with mild or moderate Alzheimer disease and 15 controls by proton spectroscopy with the voxel located in the posterior cingulate. The MMSE was applied to all patients and controls. The metabolic ratios: N-acetyl-aspartate/creatine (Naa/Cr), mio-inositol/creatine (mI/Cr) and mio-inositol/N-acetyl-aspartate (mI/Naa) were obtained and then post-processed using the MRUI software (magnetic resonance user interface).

**Results:**

Correlation between Naa/Cr and mI/Naa ratios in the posterior cingulate with the MMSE was observed, and a positive correlation with Naa/Cr and negative correlation with mI/Naa were seen. The mI/r ratio presented no correlation with MMSE scores.

**Conclusion:**

The positive correlation with Naa/Cr, and negative correlation with mI/Naa may corroborate that neuronal density/viability is associated to a higher MMSE score.

## Abstract (pt)

Comparar as razões dos metabólitos obtidas no giro do cíngulo posterior e os escores do Mini-Exame do Estado Mental (MEEM) em pacientes com doença de Alzheimer leve e moderada em controles cognitivamente normais.

**Métodos:**

Nós avaliamos 29 pacientes com doença de Alzheimer leve e moderada e 15 controles utilizando espectroscopia de prótons com volume de interesse único localizado no cíngulo posterior. Os escores do teste MEEM foram aplicados em todos os pacientes e controles. As razões dos metabólitos N-acetil-aspartato/creatina (Naa/Cr), mio-inositol/creatina (mI/Cr) e mio-inositol/-acetil-aspartato (mI/Naa) foram obtidas e pós-processadas usando o software MRUI (magnetic resonance user interface).

**Resultados:**

Foram encontradas correlações entre as razões Naa/Cr e mI/Naa obtidas no cíngulo posterior e os escores do teste MEEM, sendo a correlação positiva com o Naa/Cr e negativa com a razão mi/Naa. A razão mio-inositol/creatina não teve correlação com os escores do teste MEEM.

**Conclusão:**

A correlação positiva entre o Naa/cr e negativa entre o mI/Naa podem corroborar a hipótese de que a densidade/viabilidade neuronal está associada a maiores escores no teste MEEM.

Alzheimer disease (AD) is the most common cause of dementia with a relative frequency which increases with age. Conventional magnetic resonance imaging (MRI) may not detect abnormalities until late in the course of the disease.^1^

Newer techniques such as proton magnetic resonance spectroscopy (1H-MRS), which allows noninvasive assessment of some metabolites *in vivo*, can be used to detect abnormalities earlier in the disease.

In Alzheimer disease (AD) N-acetil aspartate concentration or N-acetyl-aspartate/creatine (Naa/Cr) ratio are decreased while mio-inositol concentration, mio-inositol/creatine (mI/Cr) and mio-inositol/N-acetyl-aspartate/creatine (mi/Naa) ratios are increased.^2^ Both the hippocampus and posterior cingulate are the limbic regions primarily affected in AD.

Cognitive tests are commonly employed to assess such patients, of which the MMSE is the most widely used.

The aim of the present study was to assess correlation between metabolic ratios obtained by proton spectroscopy in the posterior cingulate, with MMSE scores in patients with mild to moderate AD and in cognitively normal controls.

## Methods

This was a prospective study based on proton spectroscopy analysis carried out in the Magnetic Resonance section of Hospital das Clinicas of the University of São Paulo School of Medicine, between October 2003 and March 2005, in 45 patients drawn from the Behavioral and Cognitive Neurology outpatient unit of Hospital das Clinicas, and from the Cognitive Disorders Reference Center (CEREDIC).

This project was approved by the Ethics committee of the Hospital das Clinicas of the University of São Paulo School of Medicine, where patients or their legal guardians signed the free informed consent term after agreeing to participate in the study.

The inclusion criteria of the study were: patients with diagnosis of probable AD according to the NINCDS-ADRDA criteria, and mild to moderate dementia according to the DSM-III R criteria, signing of the informed consent term by the patient or their legally responsible guardian, and a collaborative patient.

All patients underwent tests for levels of Vitamin B12, thyroid hormones, serology for syphilis, hemogram, levels of urea and creatinine, hepatic enzymes, total fraction proteins, and magnetic resonance imaging (MRI) examination to rule out other causes of cognitive deficits.

Exclusion criteria applied were: patients with psychiatric or neurologic diseases, history of cranial trauma, use of psychotropic medication (except drugs for treatment of AD), *diabetes mellitus*, evidence of focal or diffuse brain lesions such as tumors, hydrocephalus or cerebral infarcts on MRI. The presence of sparse focus of high signal in the white matter of cerebral hemispheres on T2-weighted sequences did not constitute exclusion criteria given that these are commonly observed in elderly patients (class 1 and 2 on the Fazekas and Schmidt scale);^3,4^ spectroscopies were excluded if after homogenization of the magnetic field, the value of the water peak width in frequency units (FWHM – *frequency width at half maximum*) exceeded 7 Hz.

Based on these criteria, 16 patients were excluded (11 with diabetes mellitus, four due to cerebral infarcts and one due to *diabetes mellitus* and meningioma), giving a final study sample of 29 patients. Age ranged from 56 to 87 years (mean 74.2±7.^6^ years and median of 75 years). Of the 29 subjects included, 17 were female (59%) and 12 male (41%). Moreover, 23 patients had mild AD while 6 had moderate AD.

A control group of 15 volunteers was used, constituting individuals without cognitive deficits, these patients were from the general population. Control group age ranged from 66 to 79 years (mean of 72.5±3.3 years). Nine volunteers were female (60%) and 6 were male (40%).

All volunteers presented normal values on the MMSE,^5^ as well as on the delayed recall of 10 figures^6^ and on the category fluency test-animals/min,^7^ where values were adjusted for schooling when appropriate.

The MRI studies were performed on a 1.5 Tesla Unit (Horizon LX 8.3, GE Medical Systems Milwaukee, WI, USA) for all patients and controls using a brain quadrature coil. The MRI and 1 H-MRS examinations took approximately 60 minutes.

The MRI protocol included: spin echo sagittal T1-weighted, fast spin echo axial T2-weighted-images, axial FLAIR images (fluid attenuated inversion recovery), axial diffusion-weighted images, axial SPGR – spoiled gradient recalled acquisition in steady state, and localizer axial T2-weighted images for planning the spectroscopy.

The 1 H-MRS protocol included a single voxel acquisition using the PRESS (point resolved spectroscopy) technique with TR=1500 ms, TE=135 ms, field of view (FOV)=24 cm, 8 NEX (number of excitations), and 96 and 128 excitations, lasting approximately 3 minutes. The voxel was located in the posterior cingulate. Preceding the spectroscopy acquisition, automatic adjusting transmission-reception, water suppression and field homogeneity optimization were performed for the selected voxel.

**Figure 1:**
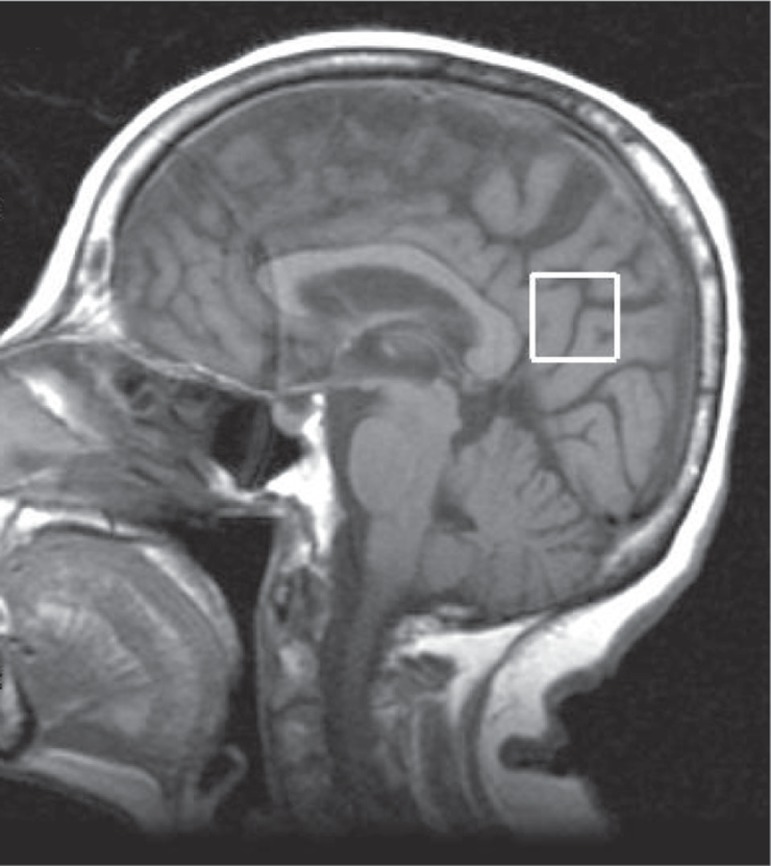
Sagittal T1-weighted image (TR=450 ms, TE=8 ms), showing voxel in the posterior cingulate.

**Figure 2:**
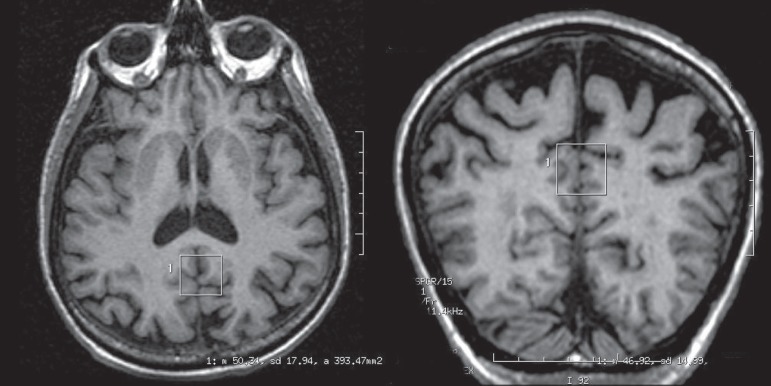
Representation of voxel in axial and coronal planes from volumetric images (SPGR).

The voxel measured 2x2x2 cm (8 cm^3^) and was located in the posterior cingulate in the median image of the saggital plane. It was positioned below the cingulate and above the parietoccipital sulcus, including the posterior cingulate as well as the inferior pre-cuneus, a location previously described in the literature^8-12^ (Figures 1 and 2).

We evaluated the N-acetyl-aspartate/creatine (NAA/CR), Mio-inosytol/creatine (mI/CR) and Mio-inosytol/N-acetyl-aspartate (mI/NAA)/ratios. Post-processing was carried out using the MRUI software (*Magnetic Resonance User Interface*), Java version, from *Advanced signal processing for medical resonance imaging and spectroscopy*, TMR, FMRX-CT97-0160. The NAA/CR, mI/CR, and mI/NAA ratios obtained in the posterior cingulate for patients and controls were compared.

Two illiterate patients (12 and 13), together with a mild AD patient (patient 14), presenting with a marked compromise in language and a very low MMSE score (MMSE=6), disproportionate to the other cognitive functions which remained relatively preserved, were excluded from this analysis. This lack of literacy and marked language impairment hampered the MMSE analysis and scoring which could have in turn introduced a bias in the association of the relationship between metabolites and the MMSE.

The Chi-square test was used to compare the gender distribution of the two groups (nominal data). The Student-t test was employed to compare the metabolite ratios in patient and control groups. Correlation between MMSE and metabolic ratios were calculated using Pearson’s linear coefficient (13). The descriptive level considered significant was 5% (<0.05).

## Results

The MMSE data was distributed as depicted in Table 1, which shows a lower MMSE and greater variance in the patient group.

**Table 1:** MMSE Scores in patients and controls.

Group	Mean	Median	SD	Minimum	Maximum	Samples
Controls	27.8	28	1.4	25	30	15
Patients	19.0	19	3.9	6	28	29

SD, standard deviation; MMSE, mini-mental state examination.

Comparative analysis between the MMSE and metabolic ratios was carried out by calculating the indexes of Pearson’s linear correlation for each metabolite, considering the entire sample and also patient and control groups separately. Separate group analysis revealed no significant association. However, analysis of the pooled data (patients and controls) revealed a significant correlation for some measures, being positive for the Naa/Cr ratio in the cingulate (0.61), and negative for the mI/Naa ratio (–0.55). These values indicated some degree of association, but evidenced large spread between the MMSE and the relationships among the metabolites. This data can be seen in Table 2.

**Table 2:** Association between MMSE and ratios among metabolites.

	Global			Patients			Controls	
Measure	Correlation	p		Correlation	p		Correlation	p
Naa/Cr	0.61	<0.001		0.27	0.176		-0,46	0,086
mI/Cr	-0,33	0,035		-0.29	0.149		-0.45	0.094
mI/Naa	-0.55	<0.001		-0.36	0.071		-0.11	0.702

Naa/Cr, N-acetil-aspartate/creatine ratio; mI/Cr, mioinositol/creatine ratio; mI/Naa, mioinositol/n-acetilaspartate ratio, and p, descriptive level.

**Figure 3:**
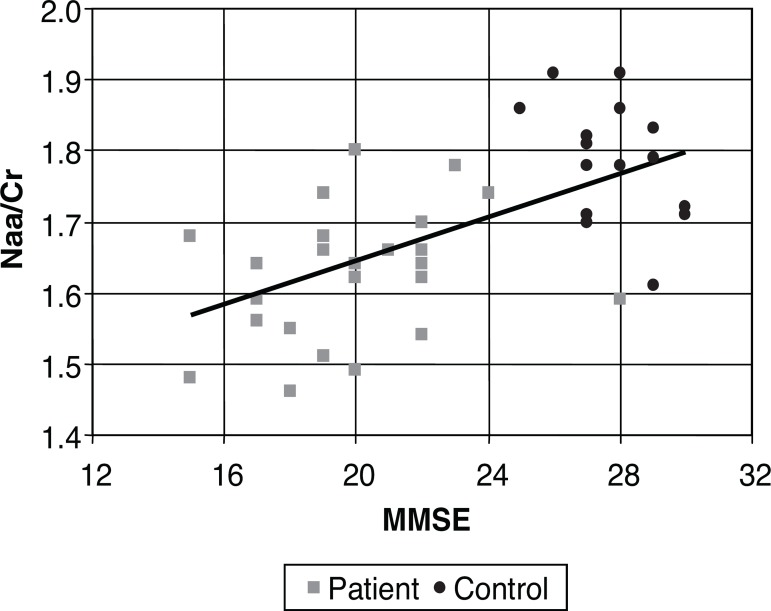
Scatter plot for MMSE and Naa/Cr in the cingulated. Naa/Cr, N-acetil-asparte/creatine ratio.

**Figure 4:**
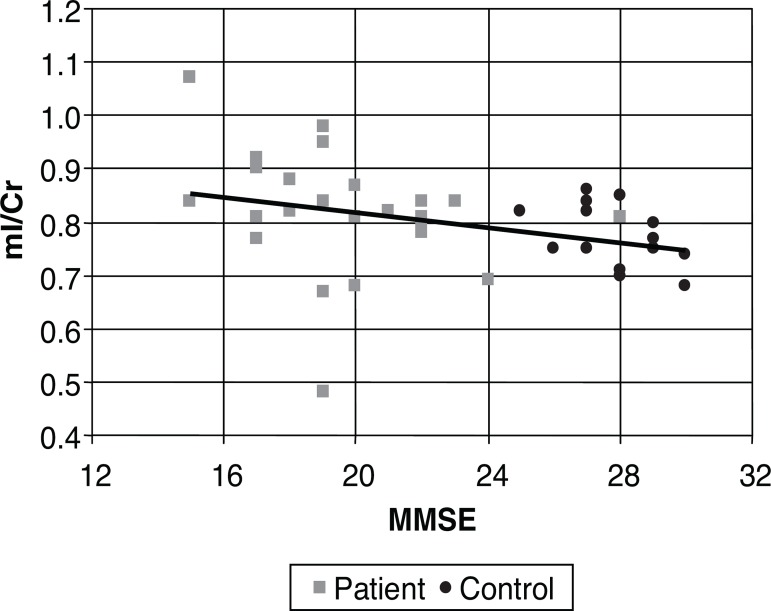
Scatter plot for MMSE and ml/Cr in the cingulate. ml/Cr, mioinositol/creatine ratio.

**Figure 5:**
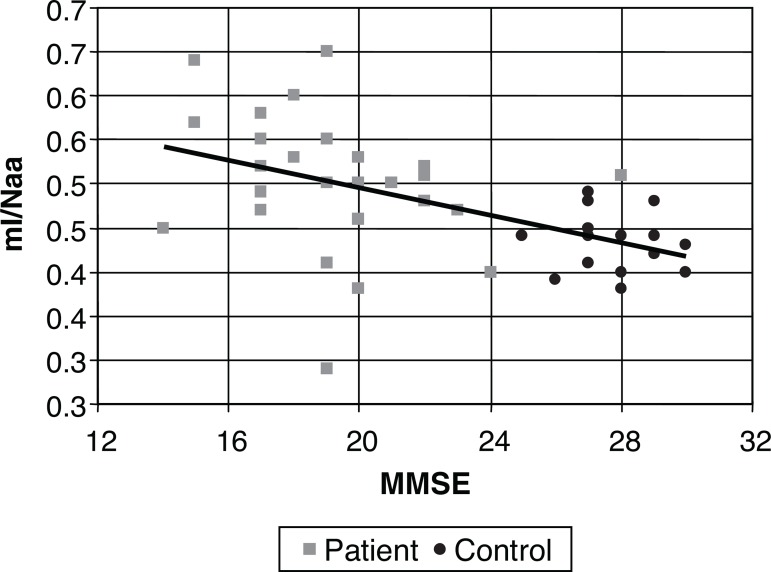
Scatter plot for MMSE and ml/Naa in the cingulate. ml/Naa, mioinositol/N-acetil-aspartate ratio.

The level of association is clearly illustrated in Figures 3 to 5, where each individual is represented by a point, and a line of correlation is presented for the global data.

## Discussion

A significant association was observed between Naa/Cr and ml/Naa ratios in the posterior cingulate and the MMSE. The Naa/Cr ratio correlated positively while the ml/Naa ratio showed negative correlation. Naa and Naa/Cr ratio values have correlated positively with the MMSE in earlier studies in the occipital and parietal gray matter,^14^ parietoccipital gray matter^15^ occipital gray matter,^16^, temporal white matter^17^ and para-hippocampal gyrus (in a post-mortem study).^18^ There is also evidence of an association between decreased Naa concentration and cognitive decline.^19,20^ Since NAA is considered a marker for neuronal density/viability, these associations indicate that the NAA/Cr ratio obtained by proton spectroscopy could be considered an indicator of cognitive decline in such patients.

The Naa/ml ratio also correlated positively with the MMSE.^15^ However, conflicting results have been found for this association regarding separate ml or ml/Cr. Several studies have demonstrated the presence of negative correlation for the ml/Cr ratio in the posterior cingulate,^21^ or for ml concentration in frontal white matter.^22^ Nevertheless, other authors^14,15^ observed no significant correlation of the ml/Cr ratio or the ml concentration, with the MMSE. It is possible that the correlation of the Naa/ml or ml/Naa is due to the Naa component. Indeed, the present study showed a more significant correlation for the Naa/Cr ratio than the ml/Naa ratio. In view of the fact that the increase in ml or the ml/Cr ratio found in this disease is early and associated with the accumulation of neurofibrillar tangles and astrocytic and glial proliferation, the absence of correlation between ml/Cr ratios and the MMSE test remains unclear and warrants future investigations involving a larger patient series.

Concluding, the analysis of proton spectroscopy studies on a single voxel within the posterior cingulate carried out in mild or moderate AD patients and controls evidenced positive correlation of the Naa/Cr ratio with the MMSE yet negative correlation of the ml/Naa ratio. These findings corroborate a correlation between neuronal density/viability and the MMSE test.
